# Advance in Surgery

**Published:** 1916-03

**Authors:** 


### Advances in Surgery.

As a result of the greatly increased number of wounded in the European war, surgeons have been making experiments that were never dreamed of a decade ago, and the results obtained are often almost marvelous. Even silver-plated ribs have been substituted for bone, and other unbelievable wonders performed. The war will prove a valuable contribution to the science of surgery, even if no other good comes from it.

